# Estimating the molecular evolutionary rates of mitochondrial genes referring to Quaternary ice age events with inferred population expansions and dispersals in Japanese *Apodemus*

**DOI:** 10.1186/s12862-015-0463-5

**Published:** 2015-09-15

**Authors:** Yutaro Suzuki, Morihiko Tomozawa, Yuki Koizumi, Kimiyuki Tsuchiya, Hitoshi Suzuki

**Affiliations:** Laboratory of Ecology and Genetics, Graduate School of Environmental Science, Hokkaido University, Sapporo, 060-0810 Japan; Department of Biology, Keio University, Yokohama, 223-8521 Japan; Laboratory of Bioresources, Ooyo Seibutsu Co. Ltd., Tokyo, 107-0062 Japan

## Abstract

**Background:**

Determining reliable evolutionary rates of molecular markers is essential in illustrating historical episodes with phylogenetic inferences. Although emerging evidence has suggested a high evolutionary rate for intraspecific genetic variation, it is unclear how long such high evolutionary rates persist because a recent calibration point is rarely available. Other than using fossil evidence, it is possible to estimate evolutionary rates by relying on the well-established temporal framework of the Quaternary glacial cycles that would likely have promoted both rapid expansion events and interisland dispersal events.

**Results:**

We examined mitochondrial cytochrome *b* (*Cytb*) and control region (CR) gene sequences in two Japanese wood mouse species, *Apodemus argenteus* and *A. speciosus*, of temperate origin and found signs of rapid expansion in the population from Hokkaido, the northern island of Japan. Assuming that global warming after the last glacial period 7–10 thousand years before present (kyr BP) was associated with the expansion, the evolutionary rates (sites per million years, myr) of *Cytb* and CR were estimated as 11–16 % and 22–32 %, respectively, for *A. argenteus*, and 12–17 % and 17–24 %, respectively, for *A. speciosus*. Additionally, the significant signature of rapid expansion detected in the mtDNA sequences of *A. speciosus* from the remaining southern main islands, Honshu, Shikoku, and Kyushu, provided an estimated *Cytb* evolutionary rate of 3.1 %/site/myr under the assumption of a postglacial population expansion event long ago, most probably at 130 kyr BP. Bayesian analyses using the higher evolutionary rate of 11–17 %/site/myr for *Cytb* supported the recent demographic or divergence events associated with the Last Glacial Maximum. However, the slower evolutionary rate of 3.1 %/site/myr would be reasonable for several divergence events that were associated with glacial periods older than 130 kyr BP.

**Conclusions:**

The faster and slower evolutionary rates of *Cytb* can account for divergences associated with the last and earlier glacial maxima, respectively, in the phylogenetic inference of murine rodents. The elevated evolutionary rate seemed to decline within 100,000 years.

## Background

To investigate the recent evolutionary history of organisms, many phylogeographic studies have assessed molecular dating using rapidly evolving markers, such as mitochondrial DNA (mtDNA) gene sequences. However, a gap has recently been suggested to exist in the evolutionary rates of mtDNA gene sequences between interspecific divergence and intraspecific polymorphism and, therefore, the assumption that a single molecular clock applies throughout all lineages over time may not be correct (e.g. [1–4]). In fact, to analyze evolutionary history on a recent timescale, substantially higher rates [*e.g.*, 38.9 or 40 %/site/million years (myr)] in the mtDNA control region (CR) have been proposed in studies of rodents such as the field vole (*Microtus agrestis* [5]) and house mouse (*Mus musculus* [6]), based on paleoclimatological and/or geological events using Bayesian inferences, contrary to the rates “traditionally” used for mammals (*e.g.*, 2*–*4 %/site/myr; [7, 8]). However, how long such a high evolutionary rate is applicable [9, 10] remains unclear due to the lack of reliable calibration points, such as fossil evidence below the species level.

*Apodemus* species in the Japanese Islands are ideal subjects for addressing this issue. In Eurasia, wood mice (*Apodemus* spp.) are widely distributed throughout temperate and subarctic regions, and their evolution has been spurred by habitat topography and the expansion of the temperate zone in the Tertiary period [11–14]. Two endemic *Apodemus* species in Japan (*A. argenteus* and *A. speciosus*) are known to have a long (5–6 myr) evolutionary history, independent from that of congeneric continental species according to molecular phylogenetic studies [14, 15]. Because both of these species are common in temperate habitats, such as broadleaf forests, their demography appears to be associated with the reduction and expansion of temperate environments during the Quaternary glacial cycles, as suggested in previous phylogeographic studies [16, 17]. Thus, one can estimate a molecular evolutionary rate by associating a specific glacial cycle with a sign of population size change in a phylogenetic diversification pattern. Recent studies on pollen stratigraphy, for example, revealed that the reduction in temperate broadleaf forest during the Last Glacial Maximum (LGM) was severe in the northern Japanese Islands, such as Hokkaido and northern Honshu [18, 19]. Thus, we may be able to use this event as a calibration point to estimate a molecular evolutionary rate over a short timescale.

Another advantage of studying these two *Apodemus* species is that we can use information on the timing of gene flow among isolated island populations to evaluate the estimated evolutionary rates. Both species inhabit almost all Japanese Islands. The Japanese Islands comprise the four main islands of Hokkaido, Honshu, Shikoku, and Kyushu arranged from northeast to southwest, as well as several insular regions, such as Sado Island, the Izu Islands, the Tsushima Islands, and the Satsunan Islands. Some of these islands are separated from the main islands by deep sea straits (depth 100–200 m) that seem to have limited the dispersal of terrestrial animals except when the sea level dropped during Quaternary glacial periods. In particular, the populations of *A. speciosus* on peripheral islands will provide useful information. For example, the divergence time between the Hokkaido and Honshu populations of *A. speciosus* is similar to those of other island populations isolated by deep sea straits, suggesting that colonization events into the peripheral islands occurred simultaneously when the sea level dropped in the Quaternary glacial periods [16, 17, 20]. Such *a priori* information about periodic gene flow to isolated island populations will help in evaluating the validity of the evolutionary rate estimates.

In this study, we focused on the postglacial population expansion of the two *Apodemus* species in Hokkaido, and then estimated the evolutionary rates of two mtDNA sequences, cytochrome *b* (*Cytb*) and CR, the most popular markers for phylogenetic and phylogeographic inferences in mammals. One can reasonably presume that the two species, which are of temperate origin, would have been affected by the Quaternary glacial periods [21], and that the population in Hokkaido, the northernmost main island of Japan, would have been especially impacted. We also evaluated the validity of the estimated evolutionary rates by comparing the sequence divergence time calculated using the evolutionary rates and the temporal aspects of the divergence events between the four main islands and peripheral islands, which are associated with periodic changes in sea level in the straits.

## Methods

### Sample collection

**Fig. 1:**
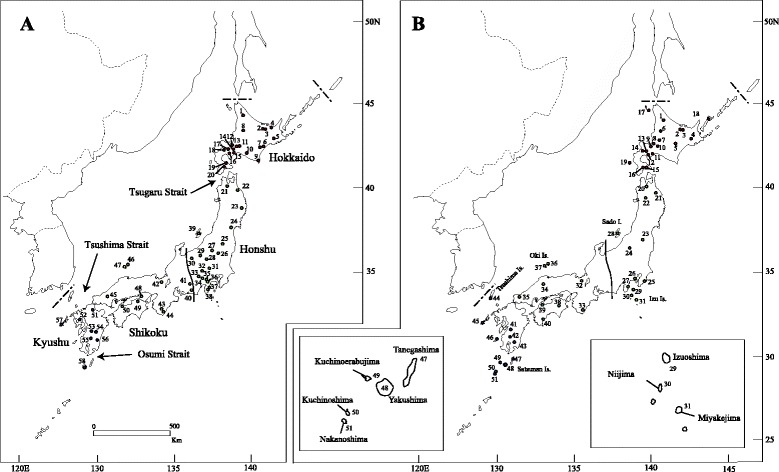
Collection locations for (**a**) *Apodemus argenteus* and (**b**) *A. speciosus*. The Japanese islands were divided into five geographical regions, Hokkaido, Eastern Honshu, Western Honshu, Shikoku, and Kyushu. The solid line indicates the border of the karyotype forms of eastern (2n = 48) and western (2n = 46) Honshu in *A. speciosus*. Circles indicate collection locations. Numbers with circles correspond to the locality number in Table 1

In total, we used 134 *A. argenteus* individuals (58 localities) and 128 *A. speciosus* individuals (51 localities) (Fig. 1, Table 1). The samples included DNA and tissue samples stored in our laboratory [14, 16], in addition to new specimens from Hokkaido, collected between 2011 and 2013. These animals were captured specifically for this study, and appropriate permissions were obtained from all relevant prefectural authorities. All experiments involving the sacrifice of live animals captured in the field were conducted within the ethical guidelines of the Mammal Society of Japan.

**Table 1:** Specimens of *Apodemus argenteus* and *A. speciosus* assessed for cytochrome *b* and control region sequences

Collection locality		n	HS codes		Collection locality	n	HS codes
*Apodemus argenteus*				*Apodemus speciosus*			
Hokkaido				Hokkaido			
1	Otoineppu	6	4089, 4839, 4840, 4845–4847	1	Nayoro	2	3922, 3923
2	Misato, Kitami	2	5116, 5117	2	Misato, Kitami	2	5118, 5120
3	Nikoro, Kitami	5	5122–5126	3	Nikoro, Kitami	3	5114, 5115, 5127
4	Koshimizu	2	5259, 5262	4	Shibecha	8	5263–5270
5	Shibecha	1	5271	5	Otofuke	2	2656, 2783^b^
6	Urahoro	1	3911	6	Fukagawa	3	2218, 3318, 3321
7	Makubetsu	2	4252, 4848	7	Bibai	1	97
8	Fukagawa	2	3313, 3314	8	Tobetsu	1	4147
9	Erimo	1	5044	9	Mt. Teine, Sapporo	2	3667, 3672
10	Hidaka	4	4841–4844	10	Naganuma	3	238, 240^a^, 242
11	Naganuma	4	223–226	11	Tomakomai	1	5136
12	Sapporo	6	2778^b^, 3666, 3668–3670, 4838	12	Date	7	5129–5135
13	Kitahiroshima	9	4829–4837	13	Kimobetsu	2	4906, 4907
14	Chitose	1	5042	14	Rankoshi	1	3836
15	Tomakomai	1	5055	15	Hakodate	1	4547
16	Date	11	5043, 5045–5054	16	Hokuto	1	5138
17	Kimobetsu	2	4904, 4905	17	Rishiri Is.	6	252–257
18	Rankoshi	1	2388	18	Kunashiri Is.	1	1103
19	Onuma	3	361^a^, 362, 433	19	Okushiri Is.	9	209–217
20	Hokuto	8	5056–5063	Eastern Honshu			
Eastern Honshu				20	Aomori, Aomori Pref.	1	389
21	Aomori, Aomori Pref.	1	392	21	Karumai, Iwate Pref.	1	375
22	Hachinohe, Aomori Pref.	1	294	22	Mt. Moriyoshi, Akita Pref.	1	301
23	Kamihei, Iwate Pref.	1	662	23	Koriyama, Fukushima Pref.	1	127
24	Miyagi Pref.	1	3288	24	Oze, Gunma Pref.	1	303
25	Koriyama, Fukushima Pref.	1	284	25	Katsuura, Chiba Pref.	1	378
26	Nikko, Tochigi Pref.	2	82, 83	26	Yokohama, Kanagawa Pref.	1	22
27	Oze, Gunma Pref.	1	295	27	Mt. Amagi, Shizuoka Pref.	1	189
28	Mt. Haruna, Gunma Pref.	2	153, 158	28	Sado Is.	9	100, 101, 104, 2803, 2804, 2805–2808
29	Shiga, Nagano Pref.	1	2158	29	Izuoshima Is.	1	181
30	Takasegawa, Nagano Pref.	1	1250	30	Niijima Is.	2	3259^b^, 3260^b^
31	Chichibu, Saitama Pref.	1	88	31	Miyakejima Is.	2	49^b^, 51
32	Odarumi, Yamanashi Pref.	2	4851, 4852	Western Honshu			
33	Minami-Alps, Yamanashi Pref.	1	4849	32	Ashiu, Kyoto Pref.	1	282
34	Aokigahara, Yamanashi Pref.	1	285	33	Mt. Nachi, Wakayama Pref.	1	349
35	Subashiri, Shizuoka Pref.	1	424	34	Hiwa, Hiroshima Pref.	1	73
36	Gotemba, Shizuoka Pref.	1	4850	35	Akiyoshidai, Yamaguchi Pref.	1	3014
37	Ito, Shizuoka Pref.	1	1217	36	Dogo, Oki Is.	1	172
38	Mt.Amagi, Shizuoka Pref.	2	187^b^, 191	37	Dozen (Nishinoshima), Oki Is.	4	173–176
39	Ryotsu, Sado Is.	2	102, 105^b^	Shikoku			
Western Honshu				38	Mt. Tsurugi, Tokushima Pref.	1	310
40	Hamamatsu, Shizuoka Pref.	3	1433^b^, 4542, 4543	39	Mt. Narabara, Ehime Pref.	2	5320, 5321
41	Mt. Chausu, Aichi Pref.	1	1438	40	Saga, Kochi Pref.	1	53
42	Ashiu, Kyoto Pref.	2	289, 290^b^	Kyushu			
43	Tanabe, Wakayama Pref.	2	35, 37	41	Mt. Shiragami, Kumamoto Pref.	1	143
44	Mt. Nachi, Wakayama Pref.	4	350, 4825–4827	42	Ebino, Miyazaki Pref.	1	308
45	Yoshika, Shimane Pref.	1	142	43	Miyakonojo, Miyazaki Pref.	2	3002^b^, 3003 ^b^
46	Dogo, Oki Is., Shimane Pref.	3	177^b^, 178, 179	44	Tsushima Is.	7	68^b^, 69–72, 94, 309
47	Dozen, Oki Is., Shimane Pref.	1	180^b^	45	Fukuejima Is.	1	3010^b^
Shikoku				46	Kamikoshikijima Is.	1	277^b^
48	Shodoshima Is.	1	135^b^	47	Tanegashima Is.	11	107–116, 117
49	Shioe, Kagawa Pref.	2	1147^b^, 1216^b^	48	Yakushima Is.	1	28
50	Mt. Narabara, Ehime Pref.	6	5314–5319	49	Kuchinoerabujima Is.	5	1176, 2809, 3017^b^, 3018^b^, 3080
-	Shikoku (exact locality unknown)	1	1058	50	Kuchinoshima Is.	3	1177–1179
Kyushu				51	Nakanoshima Is.	4	14, 98, 1180, 1181
51	Soeda, Fukuoka Pref.	1	360				
52	Oomura, Nagasaki Pref.	1	3026^b^				
53	Mt. Shiragami, Kumamoto Pref.	1	292^b^				
54	Shiiba, Miyazaki Pref.	1	1919				
55	Ebino, Miyazaki Pref.	2	44, 45				
56	Miyazaki, Miyazaki Pref.	2	1807, 1811				
57	Fukuejima Is.	1	3027^b^				
58	Yakushima Is.	2	29, 31^b^				

Sequences were obtained from the DNA databases (Refs [14]^a^ and [16]^b^). Underlined DNA codes are absent in the sequence of the control region

### Sequencing analysis

DNA was extracted using a standard phenol-chloroform extraction method [22]. For both species, two mtDNA sequences were analyzed: the entire coding region of *Cytb* (1,140 bp) and half (the hypervariable region I) of CR (*A. argenteus*, 560 bp; *A. speciosus*, 554 bp). Sequence determination for *Cytb* was performed with semi-nested polymerase chain reaction (PCR), amplifying two fragments (“upper” and “lower”) in the second PCR [23]. The first run was done using the primers L14724 and H15915 [24], at a final concentration of 0.05 pmol/μl, and Amplitaq-Gold DNA polymerase (Applied Biosystems) under thermal cycling parameters of 95 °C for 10 min and 30 cycles at 95 °C for 30 s., 50 °C for 30 s., and 60 °C for 30 s. The second PCR was done with primers at 0.05 pmol/μl for both upper (R-L14724: 5′-CAGGAAACAGCTATGACCGATATGAAAAACCATCGTTG-3′, SNH655: 5′-TGTAAAACGACGGCCAGTTGTGTAGTATGGGTGGAATGG-3′) and lower (SNL497: 5′-CAGGAAACAGCTATGACC CCTAGTAGAATGAATCTGAGG-3′, R-H15916: 5′-TGTAAAACGACGGCCAGTGTCATCTCCGGTTTACAAGA-3′) regions under conditions of 30 cycles at 96 °C for 30 s, 50 °C for 60 s, and 60 °C for 30 s, using Amplitaq DNA polymerase (Applied Biosystems). PCR for the CR sequences was performed with the AmpliTaq Gold 360 Master Mix kit (Applied Biosystems) using primers L15399 (5′-GCACCCAAAGCTGATATTCT-3′; [25]) and CR1 (5′-CATGCCTTGACGGCTATGTT-3′; [23]) at a final concentration of 0.5 pmol/μl, using PCR conditions of 96 °C for 10 min and 35 cycles at 96 °C for 30 s, 50 °C for 60 s, and 72 °C for 60 s. The PCR products were sequenced using the PRISM Ready Reaction DyeDeoxy Terminator Cycle Sequencing Kit (Applied Biosystems) and an ABI3130 automated sequencer (Applied Biosystems). The sequences of both strands were determined using the universal primers (M13RP1 and −21 M13; Applied Biosystems) in *Cytb* and the primers used for PCR in CR. The sequences were aligned using ClustalW implemented in MEGA5 [26].

### Phylogenetic analysis

Phylogenetic trees with the concatenated mtDNA sequences of *Cytb* and CR were generated with MEGA5 [26], using maximum-likelihood (ML), maximum-parsimony (MP), and neighbor-joining (NJ). All indels were excluded from the phylogenetic analysis. In addition to sequences of *Apodemus peninsulae* (HS123; collected from Hokkaido), *A. speciosus* (HS97) and *A. argenteus* (HS223) were used as outgroup taxa in the phylogenetic analyses of *A. argenteus* and *A. speciosus*, respectively. In the ML analysis of the *A. argenteus* data, the Tamura three-parameter model (TN92 + G + I) was selected using MEGA5 with likelihood-ratio tests and the Akaike information criterion (AIC)*.* The Hasegawa–Kishino–Yano model (HKY85) was used in the ML tree of *A. speciosus*. In the NJ analysis, the Kimura two-parameter model (K2) was used. The reliability of nodes was assessed using 1000 bootstrap replicates. Median-joining networks were constructed for both markers using the Network program (ver. 4.6.1.2) [27]. We used ARLEQUIN (ver. 3.5) to calculate the nucleotide (π) and haplotype (*Hd*) diversity using all data [28].

To detect rapid population expansion, mismatch distribution analyses, neutrality tests (Tajima’s *D* [29] and Fu’s *F*_S_ [30]) were performed using ARLEQUIN (ver. 3.5). The significance of neutrality was tested with 1,000 replicates of coalescent simulation. The mismatch distributions of the *Cytb* sequence were calculated for all data and for each phylogroup of both species. The mismatch distributions of the CR sequences were calculated for all populations and the Hokkaido populations of each species. A smooth, unimodal distribution indicates population growth [31]. The expected distribution was simulated under the sudden expansion model [32, 33]. We tested the validity of the sudden expansion model using a parametric bootstrap approach with 1,000 replicates. In this method, for each replicate, the sum of squared deviation (*SSD*) between the observed and expected distributions is compared with the *SSD* between the simulated and expected distributions using ARLEQUIN (ver. 3.5). We used the raggedness index (r) [34] as a test statistic for the estimated sudden expansion models.

The expansion parameter tau (τ) was estimated using ARLEQUIN (ver. 3.5) [28] in each cluster in which signs of sudden demographic expansion were evident. The evolutionary rates of the mtDNA markers were estimated using the formula *t* = τ/2*u*, where *t* is the time since expansion (in generations) and *u* is the cumulative evolutionary rate per generation for the whole sequence [31]. The value of *u* was derived from the formula *u* = *μkg*, where *μ* is the evolutionary rate per site per year, *k* is the sequence length, and *g* is the generation time in years. We assumed the average generation times of both species were 1 year because both species are believed to breed once or twice per year. The time since expansion (*t*) for the Hokkaido populations of both species was assumed to be 7–10 kyr BP, based on the timing of the recovery of the *Quercus* population in northern Hokkaido from the impact of the last glacial period [19].

The time to the most recent common ancestor (TMRCA) of the *Cytb* sequences and 95 % highest posterior density (HPD) were estimated using BEAST (ver. 1.7.5; http://beast.bio.ed.ac.uk) [35]. The analysis was carried out with the HKY85 substitution model and the strict-clock model using expansion growth as the tree prior. Two European species of *Apodemus sylvaticus* (accession no. JF819979) and *Apodemus flavicollis* (accession no. JF819969) whose divergence time was estimated at 2–3 million years ago [15], were used as outgroup taxa. Bayesian searches were conducted using the Markov chain Monte Carlo (MCMC) method for 10 million generations. The first 1 million generations were discarded as a burn-in. Independence among samplings was confirmed for each run by checking for high effective sample sizes (>200). Tracer (ver. 1.5) [36] was used to assess convergence of the MCMC chains.

## Results

### Sequence data

Sequence diversity indices for the two gene data sets are summarized in Table 2. Complete *Cytb* sequences (1,140 bp) were obtained from 134 and 128 specimens of *A. argenteus* (187 variable sites that defined 101 haplotypes) and *A. speciosus* (185 variable sites that defined 83 haplotypes), respectively. No deletion or insertion was detected in the *Cytb* sequences of either species. CR sequences for *A. argenteus* (560 bp; 134 specimens) and *A. speciosus* (554 bp; 123 specimens) contained 100 and 96 variable sites that defined 91 and 78 haplotypes, respectively. In *A. argenteus*, 11 indels were detected, whereas only one insertion was detected in *A. speciosus*.

**Table 2:** Genetic diversity indices of two mitochondrial DNA markers (*Cytb* and CR) for two Japanese *Apodemus* species

Species	Gene	Phylogroup	N	*S*	*h*	π (%)	*Hd*	Tajima’s D	Fu’s Fs
*A. argenteus*									
	Cytb	I + II	134	187	101	1.09	0.980	−2.034*	−24.142**
	(1,140 bp)	I	124	160	91	0.78	0.98	−2.283**	−24.535**
		II	10	41	10	1.01	1.000	−1.02	−3.1*
		Ia	112	127	79	0.57	0.971	−2.382**	−25.001**
		Ia-1	72	59	44	0.21	0.933	−2.668**	−26.894**
		Ia-2	16	29	11	0.39	0.933	−2.015*	−14.361**
	CR	I + II	134	100	91	1.77	0.963	−1.522*	−24.443*
	(560 bp)	I	124	87	81	1.38	0.957	−1.613*	−24.713**
		II	10	34	10	2.16	1.000	−0.394	−2.974*
		Ia	112	71	70	1.09	0.95	−1.771*	−25.111**
		Ia-1	72	31	33	0.42	0.874	−2.04*	−26.995**
*A. speciosus*									
	Cytb	I + II	128	185	83	1.61	0.990	−1.415*	−23.917*
	(1,140 bp)	I	35	99	32	1.01	0.993	−1.9*	−24.526**
		II	93	114	51	1.100	0.972	−1.39	−24.268**
		IIa	56	52	29	0.41	0.942	−1.966*	−14.661**
		IIb	9	12	3	0.49	0.556	1.283	−4.484*
		IIc	4	13	4	0.67	1.000	0.82	0.02
		IId	24	46	15	0.96	0.949	−0.360	−15.999**
		IIa-1	39	29	19	0.24	0.904	−2.049*	−26.442**
		IIa ^ex_IIa-1^	17	25	10	0.53	0.868	−0.767	−13.245**
	CR	I + II	123	96	78	1.43	0.991	−1.613*	−24.685*
	(554 bp)	I	32	61	27	1.44	0.986	−1.611*	−24.931**
		II	91	64	51	1.09	0.966	−1.572*	−25.189**
		IIa	56	33	31	0.653	0.927	−1.672*	−26.077**
		IIb	8	8	3	0.69	0.607	1.1	−4.793*
		IIc	4	10	4	1.09	1.000	2.21	−0.29
		IId	23	25	13	1.14	0.941	−0.39	−21.246**
		IIa-1	39	20	19	0.330	0.868	−2.044*	−27.270**
		IIa ^ex_IIa-1^	17	17	13	1.14	0.926	−0.444	−12.799**

See Figs. 3 and 4 for corresponding phylogroups of *A. argenteus* and *A. speciosus*, respectively

*N* sample size, *S* number of substitutions, *h* number of haplotypes, *π* nucleotide diversity, *Hd* haplotype diversity

*P* < 0.05 (*), *P* < 0.001 (**)

### Population genetic analyses with mtDNA

**Fig. 2:**
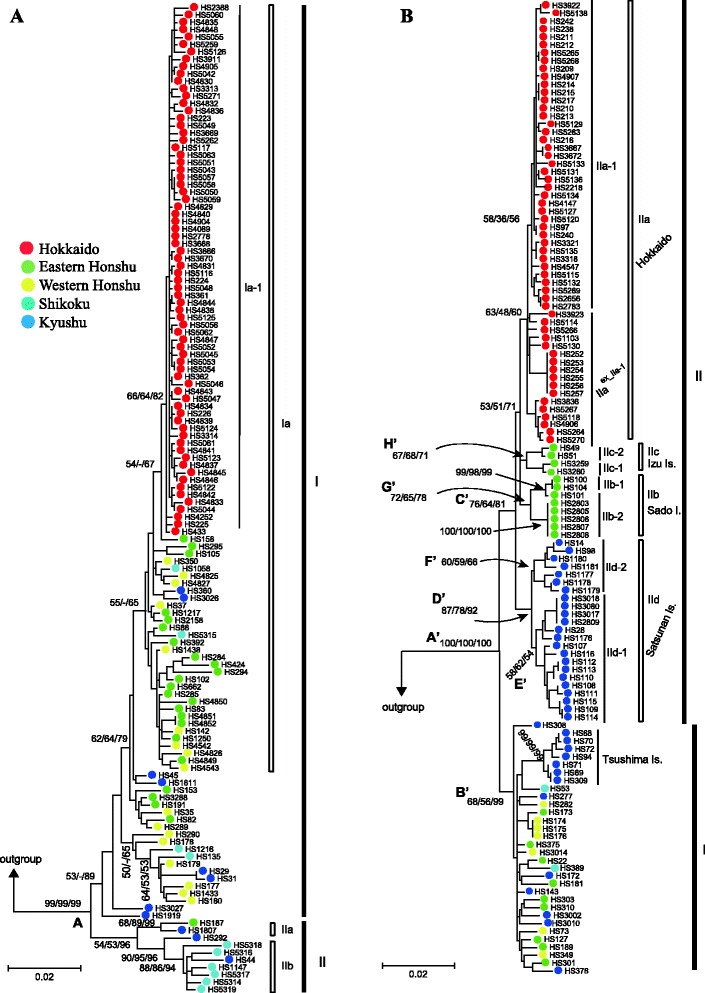
Maximum-likelihood trees for (**a**) *Apodemus argenteus* and (**b**) *A. speciosus* from concatenated mitochondrial DNA sequences (cytochrome *b* and the control region). Numbers at nodes indicate bootstrap values by the maximum-likelihood, maximum-parsimonious, and neighbor-joining methods (1000 replicates, > 50 %). The taxon names correspond to those in Table 1

**Fig. 3:**
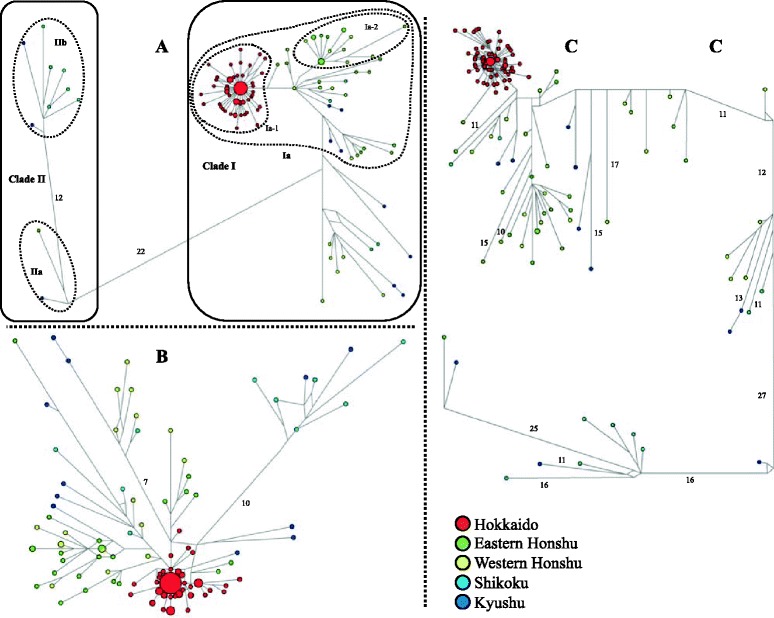
Median-joining networks constructed from (**a**) cytochrome *b* gene sequences (1,140 bp) and (**b**) control region sequences (560 bp), and (**c**) concatenated sequences (1,700 bp) for 134 *Apodemus argenteus* across Japan. The number of mutations (>10) between haplotypes is indicated in branches. The size of the circles is proportional to the number of samples. The compositions of sample localities are also reflected in each haplotype. The boxes with the solid and dashed lines are the major clusters (I, II) and clusters focused on in this study, respectively

To determine the phylogeographic structures of the two *Apodemus* species, phylogenetic trees and median-joining (MJ) network trees were constructed using *Cytb* and CR sequences, and their concatenated sequences. The phylogenetic analyses with the *Cytb* sequences and concatenated sequences of *Cytb* and CR in *A. argenteus* (Fig. 2a) revealed that the haplotypes fell into two clusters (I and II), which were supported by low (<54 % in ML and MP) and high (>89 % in NJ) bootstrap values depending the methods used, but they were unsupported (<50 %) with the CR sequences alone (data not shown). The genetic distance for the *Cytb* sequences between the two major clusters was calculated as 2.9 % on average. Cluster I contained a relatively large subcluster, representing haplotypes from almost all Japanese islands, from Hokkaido to Kyushu, with low bootstrap values (<65 %). The phylogenetic analyses further revealed a cluster representing all the Hokkaido haplotypes (Ia-1) within subclade Ia, with low to moderate supporting values (64–84 %). Notably, a haplotype from eastern Honshu (HS158 from Gunma Prefecture) clearly was closely associated with the Hokkaido cluster Ia-1, although its supporting values were low (<67 %; Fig. 2a). Cluster II was subdivided into two clusters, IIa and IIb, with relatively high supporting values. Cluster IIa contained two haplotypes from the southernmost tip of eastern Honshu (Mt. Amagi, Shizuoka Prefecture; locality 38 in Fig. 1) and Kyushu (Miyazaki, locality 56), and Cluster IIb included eight haplotypes from Shikoku and Kyushu. The MJ networks of the *Cytb*, CR, and concatenated sequences (Fig. 3) showed star-like structures for the haplotypes from Hokkaido (Ia-1), as predicted in the phylogenetic tree analysis. A portion of haplotypes from both western and eastern Honshu also showed a star-like structure (Ia-2) in the *Cytb* data set, although the structure was ambiguous in the networks of CR and concatenated data sets.

**Fig. 4:**
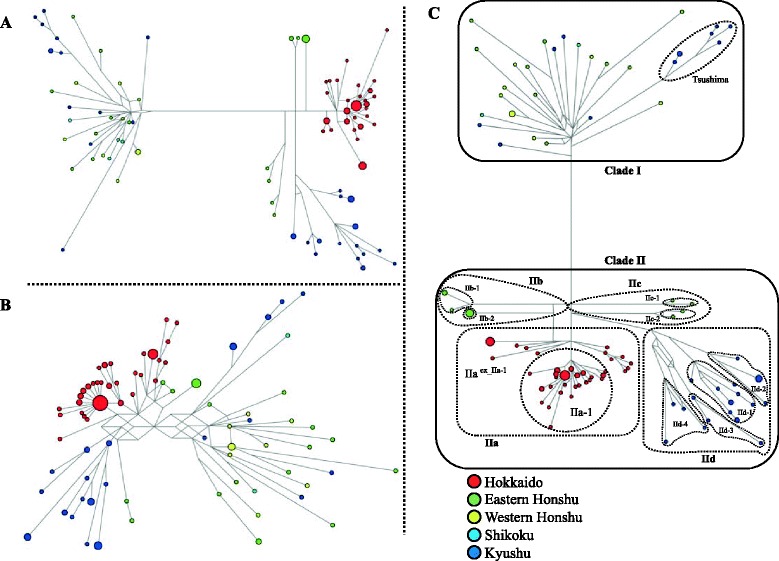
Median-joining networks constructed from (**a**) 128 cytochrome *b* gene sequences (1,140 bp) and (**b**) 123 control region sequences (554 bp), and (**c**) 123 concatenated sequences (1,694 bp) for *A. speciosus* across Japan. The number of mutations (>10) between haplotypes is indicated in branches. The size of the circles is proportional to the number of samples. The compositions of the sample localities are also reflected in each haplotype. The boxes with solid and dashed lines are the major clusters (I, II) and the clusters addressed in this study, respectively

The 83 haplotypes of the concatenated *Cytb* and CR sequences from *A. speciosus* fell into two major clusters (I and II) that were always supported, but to varying degrees depending on the methods used (Figs. 2b and 4). Cluster I included haplotypes from the three main islands of Japan (Honshu, Shikoku, and Kyushu) and their closely associated islands, such as the Oki and Tsushima Islands. The haplotypes from the Tsushima Islands were clustered together in cluster I. Cluster II encompassed four haplotype groups representing four remote island regions, Hokkaido (IIa), Sado Island (IIb), the Izu Islands (IIc), and the Satsunan Islands (IId), each of which was supported by the three tree-building methods, although the supporting values were all relatively low. The genetic distance of *Cytb* between the two clusters was 2.4 %. The MJ networks of the *Cytb* (Fig. 4a) and concatenated *Cytb* and CR sequences (Fig. 4c) showed that the clusters corresponded to those in the phylogenetic analysis of *Cytb*. The MJ network tree of the CR sequences showed no clear relationship (Fig. 4b), as was the case for *A. argenteus*. The networks of the *Cytb* and CR sequences were consistent with those from the phylogenetic tree and showed more defined relationships among the clusters (Figs. 2b and 4c). The MJ network constructed from the concatenated sequences revealed two distinct groups in the haplotypes from Hokkaido (IIa), with different levels of sequence divergence: IIa-1 (low divergence; π = 0.244 %) and IIa excluding IIa-1 (IIa^ex_IIa-1^; high divergence; π = 0.525 %). The IIa-1 group was distributed throughout Hokkaido, whereas the IIa^ex_IIa-1^ group was restricted to a few localities, such as Rishiri Island, Kunashiri Island, and the eastern part of Hokkaido. The Sado Island population (IIb) could be divided into subclusters IIb-1 and IIb-2, the Izu Islands populations (IIc) into subclusters IIc-1 and IIc-2, and the Satsunan Islands (IId) populations into subclusters IId-1 (“Osumi”) and IId-2 (“Tokara”), each of which was further subdivided into two geographic areas.

Genetic diversity indices, including the nucleotide diversity (π) and haplotype diversity (*Hd*) values, were calculated for each of the phylogroups denoted in this study (Table 2). For *A. argenteus,* the π values of the total samples were 1.091 % and 1.771 %, and the *Hd* values were 0.980 and 0.963, for *Cytb* and CR, respectively. Notably, the π values of both markers for the Hokkaido population were low (0.212 % and 0.416 %, respectively). For *A. speciosus*, the respective π values of the whole samples were 1.606 % and 1.426 %, and the *Hd* values were 0.990 and 0.991 for *Cytb* and CR, respectively. As with *A. argenteus*, the Hokkaido population showed quite low nucleotide and haplotype diversities. The Tajima’s *D* and Fu’s *F*_S_ values for the Hokkaido population of *A. argenteus* were significantly negative for both markers. In *A. speciosus,* Tajima’s *D* and Fu’s *F*_S_ values for the total sample set and the IIa-1 group were significantly negative. The IIa^ex_IIa-1^ had significant negative values for Fu’s *F*_S_ for both markers, although Tajima’s *D* was not significant. Some other clusters, such as the Ia-2 of *A. argenteus* (mainly those from the eastern part of Honshu) and cluster I of *A. speciosus*, also had significant negative values for the neutrality tests of both markers (Table 2).

**Fig. 5:**
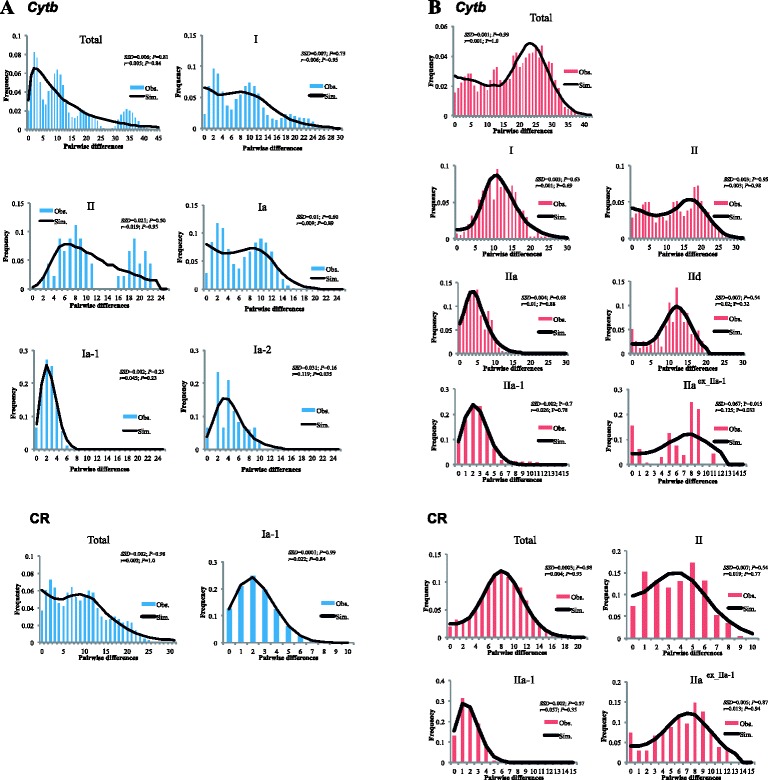
Mismatch distribution of the mitochondrial cytochrome *b* (*Cytb*) and control region (CR) sequences for *A. argenteus* (**a**) and *A. speciosus* (**b**). Bars indicate observed frequency, and a line denotes the expected frequency under the sudden expansion model. *SSD*, sum of squared deviations; *r*, Harpending’s raggedness index

The mismatch distribution analysis of the *Cytb* sequence was performed on each phylogroup except for two groups with small sample sizes (Sado Island and Izu Islands in *A. speciosus*): clusters I, II, Ia, Ia-1, and Ia-2 for *A. argenteus*, and clusters I, II, IIa, IId, IIa-1, and IIa^ex_IIa-1^ for *A. speciosus* (Fig. 5). The mismatch distribution analysis with CR sequences was conducted only for the total samples and the Hokkaido population that showed clear clustering patterns. For the majority of the clusters studied, the sudden expansion model was not rejected with either marker, but was rejected for IIa^ex_IIa-1^ of *Cytb* of *A. speciosus* (Table 3). In *A. argenteus*, analyses of the Hokkaido population showed unimodal distributions with similar τ values for both markers (τ = 2.547 and 2.482 for *Cytb* and CR, respectively). The Hokkaido population of *A. speciosus* tended to show bimodal distributions for both markers, although when separated, the two groups of Hokkaido haplotypes, IIa-1 and IIa^ex_IIa-1^, had unimodal distributions for both markers, except *Cytb* from IIa^ex_IIa-1^.

**Table 3:** Detection of rapid expansion events with the mismatch distribution analysis

Species	Marker	Cluster	*SSD (P-value)*	*r (P-value)*	*τ*	t (k years)*	μ (/site/myr)
*A. argenteus*	*Cytb*	Ia-1	0.002 (*P =* 0.25)	0.045 (*P =* 0.84)	2.55	7	16.0 %
						10	11.2 %
						15	7.5 %
	CR	Ia-1	0.0001 (*P =* 0.99)	0.022 (*P =* 0.84)	2.48	7	31.7 %
						10	22.2 %
						15	14.8 %
*A. speciosus*	*Cytb*	IIa-1	0.002 (*P =* 0.7)	0.026 (*P =* 0.78)	2.730	7	17.1 %
						10	12.0 %
						15	8.0 %
	*Cytb*	IIa ^ex_IIa-1^	0.067 (*P =* 0.015)	0.125 (*P =* 0.03)	8.57	60	6.3 %
						130	2.9 %
	*Cytb*	I	0.003 (*P =* 0.63)	0.006 (*P =* 0.69)	9.19	60	6.7 %
						130	3.1 %
	CR	IIa-1	0.002 (*P =* 0.57)	0.057 (*P =* 0.35)	1.88	7	24.2 %
						10	16.9 %
						15	11.3 %
	CR	IIa ^ex_IIa-1^	0.004 (*P =* 0.87)	0.013 (*P =* 0.94)	7.97	60	11.9 %
						130	5.6 %
	CR	I	0.004 (*P =* 0.38)	0.016 (*P =* 0.25)	8.52	60	12.8 %
						130	5.9 %

*SSD* sum of squared deviation, *r* Harpending’s raggedness index, *τ* expansion parameter, *t* time (in years) after expansion, *μ* evolutionary rate (per site per million years)

*The *t* values for the predicted relatively recent expansion event of the Hokkaido populations were set to 7,000 and 10,000 years ago based on the time frame of the recovery of the broadleaf forests in Hokkaido [19]. In addition, the possibility of expansion (15,000 years ago) immediately after the end of the LGM was also taken into account [19]

### Evolutionary rate estimation

The evolutionary rates (*μ*, per site per myr) for both markers were calculated using the formula, *t* (time after expansion, in years) = τ/2*μk* (*k*: sequence length), suggesting that the Hokkaido populations experienced rapid postglacial expansion, with the assumption that the latest expansion occurred 7–10 kyr BP, in association with the expansion of the *Quercus* population in northern Hokkaido [19]. We also accounted for the possibility that the expansion of the wood mouse population occurred immediately after the LGM (15 kyr BP), given that *Apodemus* includes habitat generalist species and that tree species in Hokkaido largely recovered after the end of the glacial maximum [19]. The evolutionary rates of *Cytb* were estimated as 7.5–16.0 %/site/myr and 8.0–17.1 %/site/myr for the Hokkaido populations of *A. argenteus* (Ia-1) and *A. speciosus* (IIa-1), respectively (Table 3). The evolutionary rates of the CR sequence were 14.8–31.7 %/site/myr and 11.3–24.2 %/site/myr for *A. argenteus* and *A. speciosus*, respectively. We estimated the evolutionary rates of *Cytb* for the predicted expansion event of cluster I of *A. speciosus* occurring in the three main islands of Honshu, Shikoku, and Kyushu, with the assumption that the expansion started from the end of the penultimate glacial time, 130 kyr BP, and obtained a value of 3.1 %/site/myr (Table 3).

### Divergence time estimation

The divergence times were estimated based on Bayesian inference using the evolutionary rates of *Cytb* obtained in the previous section. BEAST analysis was not performed with the CR sequences due to the greater inequality in branch lengths observed on the CR NJ trees compared with the *Cytb* NJ trees (data not shown), which suggested less regular substitution fixation rates over evolutionary time (*e.g.*, [15]). In the BEAST analysis for *A. argenteus* (data not shown), the divergence time between the Hokkaido population and the closest related haplotype from Honshu (HS158/Gunma Prefecture) was estimated at 39.0, 34.6, and 22.2 kyr BP, using evolutionary rates of 7.5, 11.2 and 16.0 %/site/myr, respectively.

**Fig. 6:**
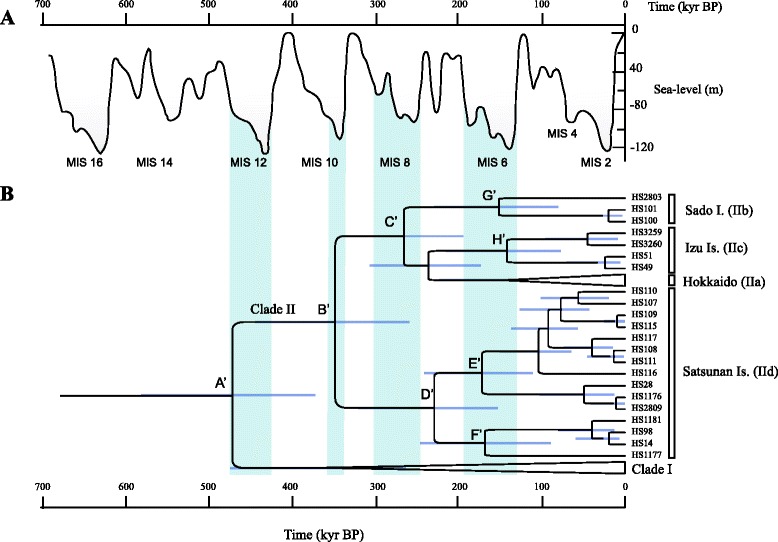
**a** Sea-level changes during the last 700 thousand years before present (kyr BP; [21]). Seven noticeable epochs of the sharp sea-level declines are marked with the stages of MIS [42]. **b** Divergence time estimates of lineages representing the four peripheral insular domains of *Apodemus speciosus*, based on a Bayesian strict molecular clock applied to the mitochondrial cytochrome *b* (*Cytb*) sequences (1,140 bp). The vertical bars indicate the 95 % highest posterior density intervals

The other older divergences addressed in this study (node A for *A. argenteus* and nodes A’–H’ for *A. speciosus*; Figs. 2 and 6) were assessed using faster evolutionary rates of 6.7, 12.0, and 17.1 %/site/myr and the rather conservative evolutionary rates reported previously, 2.4 %/site/myr [15] and 2.8 %/site/myr [13], in addition to the value of 3.1 %/site/myr. The resulting estimated divergence times, including those of the two outgroup taxa, *A. flavicollis* and *A. sylvaticus*, are summarized in Table 4.

**Table 4:** Time (in years) to the most recent common ancestor (TMRCA) for the nodes addressed in this study

Species		Lineage						TMRCA (95 % HPD)							
	Node		K2P (%)	17.1 %		12.0 %		6.7 %		3.1 %		2.8 %		2.4 %	
*A. argenteus*															
	A	Clades I/II	2.93	103	(83–133)	156	(118–188)	270	(210^335)	589	(453–729)	650	(500–809)	750	(529–938)
*A. speciosus*															
	A’	Clades I/II	2.43	83	(62–102)	120	(94–146)	214	(169–264)	463	(365–569)	512	(399–624)	595	(469–733)
	B’	Clade II	1.78	59	(44–60)	92	(66–124)	177	(115–244)	355	(252–478)	396	(276–529)	450	(325–605)
	C’	Hokkaido/Sado/Izu	1.49	46	(34–60)	67	(49–87)	120	(88–155)	260	(190–333)	290	(210–369)	332	(247–429)
	D’	Satsunan Is.	1.30	41	(27–56)	60	(39–83)	107	(70–145)	229	(153–317)	256	(168–356)	289	(200–403)
	E’	“Osumi” (Satsunan Is.)	1.01	30	(20–42)	44	(29–62)	78	(52–100)	171	(107–235)	191	(125–268)	220	(143–304)
	F’	“Tokara” (Satsunan Is.)	0.91	30	(16–44)	43	(24–65)	76	(43–113)	167	(90–245)	187	(101–277)	215	(113–315)
	G’	Sado I.	0.98	26	(14–39)	39	(22–59)	69	(36–100)	150	(78–222)	166	(82–247)	193	(102–290)
	H’	Izu Is.	0.88	25	(13–37)	36	(19–54)	65	(36–97)	139	(72–208)	156	(88–231)	175	(101–267)
Two European species															
	*A. flavicollis/A. sylvaticus*		12.8	343	(286–408)	491	(405–579)	891	(736–1044)	1899	(1553–2240)	2160	(1731–2486)	2455	(2025–2915)

Four different evolutionary rates (*μ*; per site per million years) were used. TMRCA and the 95 % highest posterior density (in parentheses) were computed with BEAST (ver. 1.7.5) [35] using the substitution model HKY and the strict clock model. The Kimura 2-parameter distance (K2P) was calculated using MEGA5 [26]. Codes for clusters and nodes are as shown in Figs. 3 and 5

## Discussion

The mtDNA phylogeographic structures of Japanese wood mice of temperate origin showed signs of rapid population expansion and lineage diversification, presumably reflecting global climatic and sea-level changes during the Quaternary glacial cycles (Fig. 6). This would thus provide calibration points for assessing evolutionary rates and an opportunity for evaluating the validity of the predicted evolutionary rates and those obtained from interspecies phylogenetic inferences using calibration points from fossils.

### Rapid expansion events of wood mice and associated climatic changes

Mismatch distribution analyses (Fig. 5) and neutrality tests (Table 2) suggested that both *A. argenteus* (τ = 2.55 for *Cytb*) and *A. speciosus* (τ = 2.73) expanded recently in Hokkaido. Considering the paleoenvironment of Hokkaido and the ecological features of the *Apodemus* species, the recent population expansions in Hokkaido most likely occurred coincidentally with rapid global warming after the LGM (~20 kyr BP). In northern Europe and North America, the present phylogeographic structures of temperate species have been influenced greatly by population size changes due to glacier extension in the LGM and the subsequent period of global warming [37]. Similarly, the phylogeographic structures of temperate species in Japan were reportedly influenced by the LGM, especially in the northern part of the islands, including northern Japan [38, 39]. The impact of the LGM and subsequent Younger Dryas glacial re-advancement (YD, 11–12 kyr BP [40]) changed the tree species composition of Hokkaido forests from cold coniferous species to temperate broad-leaved species, including oak trees (*Quercus*) [41]. Because both of the *Apodemus* species generally inhabit broad-leaved forests, the signatures of the recent expansion events of the two *Apodemus* species in Hokkaido are likely to have been associated with the expansion of the temperate broadleaf forests after the YD (~7–10 kyr BP [40]), although we can not exclude the idea that the expansion event occurred shortly after the LGM, 15 kyr BP.

The mismatch analysis and network analysis of Hokkaido *A. speciosus* populations (Figs. 4 and 5) suggested another expansion of IIa^ex_IIa-1^, with a τ of 8.57. We observed a star-like pattern for this haplotype group in the networks with the CR and concatenate sequences but not in that for *Ctyb*. Given that the expansion event for IIa-1with τ = 2.73 is most likely to have occurred 7–15 kyr BP, the other expansion event for IIa^ex_IIa-1^ is likely attributable to the next nearest global climate fluctuation (the rapid cooling and subsequent rapid warming): more specifically, the periods 60 kyr BP or 130 kyr BP, equivalent to the terminations of marine isotope stages 4 (MIS 4) and 6 (MIS 6), respectively (Fig. 6; [42]). Deciduous broad-leaved forests are known to have decreased and increased during these periods in Japan [18, 43]. We favor the latter, because the former glacial period was less severe, leading to an extreme bottleneck effect and because this view is consistent with the expansion event of cluster I of *A. speciosus*, which is most likely due to rapid warming at the end of MIS 6, as discussed later. The haplotypes belonging to the IIa^ex_IIa-1^ group tended to show limited distributions on insular and peripheral parts of mainland Hokkaido near the coast, such as Rishiri Island, Kunashiri Island, and the eastern part of Hokkaido (Fig. 2b), indicating that in Hokkaido, the environment of the inland mountainous areas severely affected temperate species during the LGM, whereas coastal areas served as refugia. In contrast, *A. argenteus* was likely severely affected by the LGM, partly because of its preference for mountainous habitats.

The neutrality tests (Table 2) and mismatch distribution analysis (Fig. 5) suggested a historical population expansion of *A. speciosus* in Honshu, Shikoku, Kyushu, and some peripheral islands, such as the Tsushima Islands (cluster I, τ = 9.19 in *Cytb*). Comparing the τ value with the Hokkaido population (τ = 2.73), which is likely attributable to the LGM as discussed above, caused us to consider that the expansion of cluster I was likely associated with a glacial cycle older than the LGM and should have happened simultaneously or slightly before the earlier expansion presumed in Hokkaido (IIa^ex_IIa-1^, τ = 8.57). Pronounced changes in regional vegetation at the beginning of the last interglacial period (128,000 years ago; MIS 5e) have been recorded in pollen-based quantitative biome reconstructions from the North Eurasian study sites [44] and central Honshu [18, 45]. Given that the postglacial appearance of *Quercus* trees is evident [18, 44, 46], the rapid expansion of *A. speciosus* (cluster I) in Honshu, Shikoku, and Kyushu is most likely due to climatic shifts in the last interglacial period. Notably, another rodent of broad-leaved forests, the Japanese giant flying squirrel *Petaurista leucogenys*, displays a similar trend of rapid expansion and mitochondrial nucleotide diversity in the northern part of its range; this expansion likely occurred at the onset of the last interglacial period [47]. In addition, cluster I expansion generated the lineage of the Tsushima Islands (Fig. 4c); this lineage is separated from Kyushu by the Tsushima Strait, which is thought to have narrowed during the penultimate glacial maximum [48]. Thus, the expansion of cluster I conceivably started 130 kyr BP, contemporaneous with the end of the penultimate glaciation.

Similarly, the mismatch distribution and neutrality test suggest glacial cycle-coupled expansion in cluster I, representing the main islands of Honshu, Shikoku, and Kyushu in *A. argenteus* (Fig. 5a, Table 2). By contrast, except for the Hokkaido subcluster, Ia-1, the network pattern of cluster I appears to have multiple subclusters (Fig. 3), indicating the involvement of multiple expansion events that are hard to assign to specific glacial cycles due to insufficient sample size. Cluster II consists of haplotypes from the southwestern part of the Japanese islands—Kyushu, Shikoku, and the southernmost tip of the Izu Peninsula (locality 38 in Fig. 1a)—suggesting effective refugia for anciently divergent mtDNA lineages during glacial periods. The presence of refugia along Honshu, Shikoku, and Kyushu has been suggested by phylogeographic studies on other mammals, such as hares [38], black bears [39], sika deer [49], flying squirrels [50], macaques [51], and voles [52].

### A temporal view of the divergences among intraspecific lineages

Relying on the evolutionary history of the wood mice discussed above, we can estimate the molecular evolutionary rates of mtDNA over different timescales. First, attributing the latest population expansion in Hokkaido to the end of the last glacial period, the evolutionary rates of *Cytb* are estimated as 7.5–16 %/site/myr and 8.0–17.1 %/site/myr for *A. argenteus* and *A. speciosus*, respectively (Table 3). Second, if we accept that the earlier expansion of the Hokkaido population of *A. speciosus* started 130 kyr BP, the rate of *Cytb* is 2.9 %/site/myr. Finally, if we assume the expansion of *A. specious* in the southern islands of Honshu, Shikoku, and Kyushu started at 130 kyr BP, the evolutionary rate is 3.1 %/site/myr, which is comparable to those obtained previously (*e.g.*, 2.8 % and 2.4 %) in the interspecies phylogenetic inferences with fossil evidence. Accordingly, these rate estimates can be categorized into faster (8–17 %) and slower (2.4–3.1 %) rates.

In *A. speciosus*, when we ran the BEAST analysis with the faster *Cytb* evolutionary rate, the resulting estimates for divergences were quite recent (*e.g.*, 25–46 kyr BP for nodes C’–H’ with 17.1 %/site/myr; Table 4). For example, the divergences among the islands of Izu, Sado, and Hokkaido (node C’) and between Kyushu and the Satsunan Islands (node D’) were 46 and 41 kyr BP, respectively, suggesting dispersals in the MIS 3 period (30–57 kyr BP). However, this would seem highly unlikely because the sea-level drop at MIS 3 is thought to have been at most around −90 m (Fig. 6). Thus, most straits, including the Osumi Strait, which separates the Satsunan Islands and Kyushu, and the Tsugaru Strait between Honshu and Hokkaido, are supposed to have been too deep and wide in MIS 3 for interisland dispersals.

In contrast, the BEAST analysis with the lower evolutionary rate of 3.1 %/site/myr (Table 4, Fig. 6) suggests that the historical dispersals of *A. speciosus* to the remote islands happened at or immediately after either MIS 6 glacial periods (130–191 kyr BP) or older ones, of 100 kyr each [21, 53]. TMRCAs for two lineages of Izu (IIc-1, IIc-2; genetic distance), and Sado (IIb-1, IIb-2), Osumi (IId-1: Tanegashima vs. Yakushima and Kuchinoerabujima), and Tokara (IId-2: Kuchinoshima vs. Nakanoshima) islands were estimated to be 125 (kyr BP; 95 % HPD = 68–192.6 kyr BP), 131.9 (69.9–201.2), 157.5 (105–216.9), and 150.5 (86.4–219.5), respectively. Notably, the case of Sado Island is consistent with the geological evidence: at 110–130 kyr BP in the last interglacial period, Sado Island was separated into two islands by a sea strait [54]. Additionally, the resulting estimates of TMRCAs of cluster I/II, cluster II, the lineages on Hokkaido/Sado/Izu, and Satsunan Islands are 463 (kyr BP; 95 % HPD = 365–569), 355 (252–478), 260 (190–333), and 229 (153–317), respectively (Table 4, Fig. 6). These timings may have been associated with the glacial periods of MIS 12 (424–478 kyr BP), MIS 10 (334–374 kyr BP), and MIS 8 (253–300 kyr BP), respectively. The sea-level drops during these periods were more than 100 m, and thus, the peripheral islands (Hokkaido, Sado, Satsunan, and Tsushima Islands) and the main islands were most likely connected or very close to each other.

Because the application of the faster evolutionary rate to relatively ancient divergences may lead to unrealistically underestimated values, as discussed above, we favor the TMRCA estimates of 589 kyr BP (95 % HPD = 453–729), 650 (500–809), and 750 (529–938) for clusters I and II of *A. argenteus* from the slower evolutionary rates of 3.1 %, 2.8 %, and 2.4 %, respectively (Table 4). Thus, it seems reasonable to propose that the ancient divergence was associated with the end of glacial periods, most likely that following MIS 16 (621–675 kyr BP). However, applying the faster evolutionary rate would be meaningful regarding a rather recent event: specifically, the divergence of the mtDNA between Hokkaido and Honshu in *A. argenteus*. From the BEAST analysis, the divergence time of the Hokkaido lineage from the most closely related haplotype of Honshu (Gunma, HS158; locality number 28 in Fig. 1a) was estimated as 22–39 kyr BP, with evolutionary rates of 7.5–16 %/site/myr. This indicates that the mtDNA of *A. argenteus* came to Hokkaido over the Tsugaru Strait (130 m) at most 39 kyr BP. The sea level of the Japan Sea during the LGM is presumed to have been ~120 m lower than that at present [48]. If we take into account the trend of hydro-isostasy, namely that the depth of the Tsugaru Strait during the LGM was not as deep as currently seen due to absence of the weight of seawater (*e.g.*, [55]), it then seems plausible to conclude that the recent migration of the mtDNA of *A. argenteus* occurred from Honshu to Hokkaido during or immediately after the LGM across a shallower, more narrow version of the Tsugaru Strait.

### Time-dependent evolutionary rates for *Apodemus* species

Emerging evidence suggests that molecular evolutionary rates calculated over a few generations may differ substantially from those calculated by phylogenetic approaches [2, 9]. This is mainly because the molecular evolutionary rate measured over a short timescale counts mutations that are slightly deleterious and thus eventually eliminated from the population by natural selection (thus reflecting the *mutation* rate), whereas that calculated over a long timescale counts mutations that are fixed in the population by natural selection or genetic drift (thus reflecting the *substitution* rate) [4, 56]. However, it is unclear how long a high evolutionary rate remains over evolutionary timescales due to the lack of information about the time of sequence divergence at relatively long timescales, such as hundreds of thousands of generations [4].

As discussed, our data provide an ideal opportunity to assess the time dependency of molecular evolutionary rates over several hundred thousand years. The probable molecular evolutionary rates for recent timescales, such as 10 kyr BP, is faster; e.g., 8–17 %/site/myr and 11–32 %/site/myr for *Cytb* and CR, respectively (Table 3). In contrast, the use of the slower evolutionary rate (*e.g.*, ~3 %/site/myr for *Cytb*) is more favorable for assessing older lineage divergences and population expansion events, 130 kyr BP or older. This in turn indicates that the evolutionary rate declines dramatically within 100 kyr. The presumed duration of the elevated evolutionary rate is shorter than those suggested for humans or insects (1–2 million years) [56, 57], and is comparable with that estimated in galaxiid fish (<200 kyr) [58] and cichlid fish (~100 kyr) [59]. Future studies on other closely related species with different population histories may help to elucidate the time dependency of molecular rates and clarify the factors determining the duration of elevated evolutionary rates.

## Conclusions

Overall, we show that the phylogeographic structures and population histories of two wood mouse species were greatly influenced by the Quaternary glacial cycles. Their population size changes and colonization events onto isolated islands were most likely associated with climatic and sea-level changes during the last and penultimate glacial cycles. Moreover, our work yielded fruitful results with respect to estimating the molecular evolutionary rates of mtDNA sequences (*Cytb* and CR), which change depending on the time that has passed over the last 100,000 years. These contributions are valuable in phylogeographic inferences, particularly for murine rodents.

## Availability of supporting data

The nucleotide sequences reported in this paper appear in the DDBJ, EMBL, and GenBank nucleotide sequence databases under accession numbers AB974692–AB975183. Sequence data files in nexus file format, together with Supplementary Information files are available for downloading from the Dryad repository: doi:10.5061/dryad.4902q.
